# Perceived Duration Increases with Contrast, but Only a Little

**DOI:** 10.3389/fpsyg.2016.01950

**Published:** 2016-12-15

**Authors:** Christopher P. Benton, Annabelle S. Redfern

**Affiliations:** School of Experimental Psychology, University of BristolBristol, UK

**Keywords:** duration, time, contrast, vision, perception, visual perception, psychophysics

## Abstract

Recent adaptation studies provide evidence for early visual areas playing a role in duration perception. One explanation for the pronounced duration compression commonly found with adaptation is that it reflects adaptation-driven stimulus-specific reduction in neural activity in early visual areas. If this level of stimulus-associated neural activity does drive duration, then we would expect a strong effect of contrast on perceived duration as electrophysiological studies shows neural activity in early visual areas to be strongly related to contrast. We employed a spatially isotropic noise stimulus where the luminance of each noise element was independently sinusoidally modulated at 4 Hz. Participants matched the perceived duration of a high (0.9) or low (0.1) contrast stimulus to a previously presented standard stimulus (600 ms, contrast = 0.3). To achieve perceptually equivalent durations, the low contrast stimulus had to be presented for longer than the high contrast stimulus. This occurred when we controlled for stimulus size and when we adjusted for individual differences in perceived temporal frequency. Further, we show that the effect cannot be explained by shifts in perceived onset and offset and is not explained by a simple contrast-driven response bias. The direction of our results is clearly consistent with the idea that level of neural activity drives duration. However, the magnitude of the effect (~10% duration difference over a 0.9–0.1 contrast reduction) is in marked contrast to the larger duration distortions that can be found with repetition suppression and the oddball effect; particularly when these may be associated with smaller differences in neural activity than that expected from our contrast difference. Taken together, these results indicate that level of stimulus-related neural activity in early visual areas is unlikely to provide a general mechanism for explaining differences in perceived duration.

## Introduction

Given that we can judge how long a stimulus or interval lasts, it is clear that we have some sense of duration. This sense of time is presumably crucial to our ability to interact with and perceive our world. However, a growing body of neuroimaging studies has yet to identify simple neural structures responsible for time processing (Bhattacharjee, 2006; Lloyd and Arstila, 2014). This seeming lack of a readily identifiable neural substrate sits well with a view in which timing behavior arises from neural structures whose primary purpose may be the processing of other aspects of sensory information. In this view, timing mechanisms are necessarily local and are consequently modality specific.

In the visual domain, one way that the issue of modality specific mechanisms has been addressed is by studies that have looked at the effect of perceptual adaptation on duration perception. Their underlying premise is that adaptation to a particular property taps into the neural processes encoding that property (Andrews, 1964; Clifford et al., 2007); in comparison to other behavioral techniques, adaptation is therefore seen to offer a relatively direct window onto neural processes, being termed the psychophysicists' microelectrode (Frisby, 1979). These adaptation studies have used simple dynamic visual stimuli and have variously implicated visual areas such as the lateral geniculate nucleus (LGN), V1 and MT as areas critical for duration perception (Burr et al., 2007, 2011; Bruno et al., 2010; Morrone et al., 2010; Curran and Benton, 2012; Latimer et al., 2014).

For example, adaptation to oscillating sine wave patterns results in duration compression for test stimuli presented at the same location as the adaptor (Johnston et al., 2006). This tight coupling between adaptor and test location occurs with very narrow adaptors (0.75° × 1°), and when adaptor and test grating orientations differ by 90° (Ayhan et al., 2009). These findings imply the involvement of a brain area containing cells with small receptive fields and orientation-independent responses—namely, the LGN. This interpretation is further supported by evidence showing that adaptation-driven duration compression occurs with adaptor temporal frequencies that are above the flicker fusion threshold, but below the high frequency cut-off for LGN cells (Johnston et al., 2008).

Whilst there are a number of conflicting findings within the visual adaptation literature, the overarching message is that the perception of duration of dynamic visual events appears driven by modality specific mechanisms (Li et al., 2015). Given the fact that adaptation to a stimulus reduces the amount of neural activity associated with that stimulus (Andrews, 1964; Clifford et al., 2007), one simple explanation for the effect of adaptation on perceived duration is that perceived duration of visual stimulus is directly driven by the neural activity elicited with that visual stimulus (Curran and Benton, 2012).

In a similar vein, the apparent duration expansion obtained with the oddball effect has also been ascribed to the level of stimulus-associated neural activity (Pariyadath and Eagleman, 2012). In the oddball effect, a stimulus, which is regularly and repeatedly presented, is then followed by a different stimulus—the oddball—which appears to last longer (Tse et al., 2004). The connection with adaptation is that the neural activity associated with the repeated stimulus is reduced, a process that seems much the same as that found with adaptation (Bartels et al., 2008). The idea is that the longer apparent duration of the oddball reflects duration compression of the repeated stimulus, the latter being driven by the suppression in neural activity. This notion, that “…increased neural activity…translates into a longer subjective duration” (Eagleman and Pariyadath, 2009, p. 1847), has been generalized from the oddball effect to explain a variety of other findings within the duration literature (Pariyadath and Eagleman, 2007; Eagleman and Pariyadath, 2009).

We can ask whether current models of duration processing could provide a relationship between neural activity and perceived duration such as that proposed above. A wide variety of mechanisms have been proposed within the duration literature and there is little consensus as to which, if any, may form the correct underlying mechanisms (Hass and Durstewitz, 2016). However, models that temporally integrate local neural activity can potentially account for a direct relationship between perceived duration and level of stimulus-associated neural activity. A good example of such a mechanism is provided by recent rise to threshold models. In these, neural activity is integrated to produce a gradually climbing rise to threshold, with the rate of climb inversely proportional to the duration being estimated (Durstewitz, 2003; Reutimann et al., 2004; Luzardo et al., 2013; Balcı and Simen, 2016; Simen et al., 2016). Potentially, this class of models can predict the duration compression that we see with adaptation—adaptation results in neural suppression, which means less activity being integrated, which means a slower rise to threshold.

One simple visual feature, in which there is a clear monotonic increase of neural activity with an increase in magnitude, is contrast. Contrast is a measure of the variability of a stimulus normalized by the luminance of its background or by its mean luminance (Pelli and Bex, 2013). Contrast is not just difference, it is proportionate difference. Those outside the vision sciences might see contrast as a simple adjunct to stimulus brightness, however this view is mistaken. Contrast is the primary description of signal strength within the discipline. This can be motivated by the fact that early visual processes (retinal gain control mechanisms) largely factor out mean luminance and deliver a signal that is dependent on stimulus contrast (Van Nes and Bouman, 1967; Shapley and Enroth-Cugell, 1984; Peli et al., 1991, 1996; Troy and Enroth-Cugell, 1993; Mante et al., 2005). The upshot of this is that post-retinal neural responses are largely independent of mean luminance (Brooks and Jung, 1973), whilst showing a profound dependence upon stimulus contrast.

**Figure 1:**
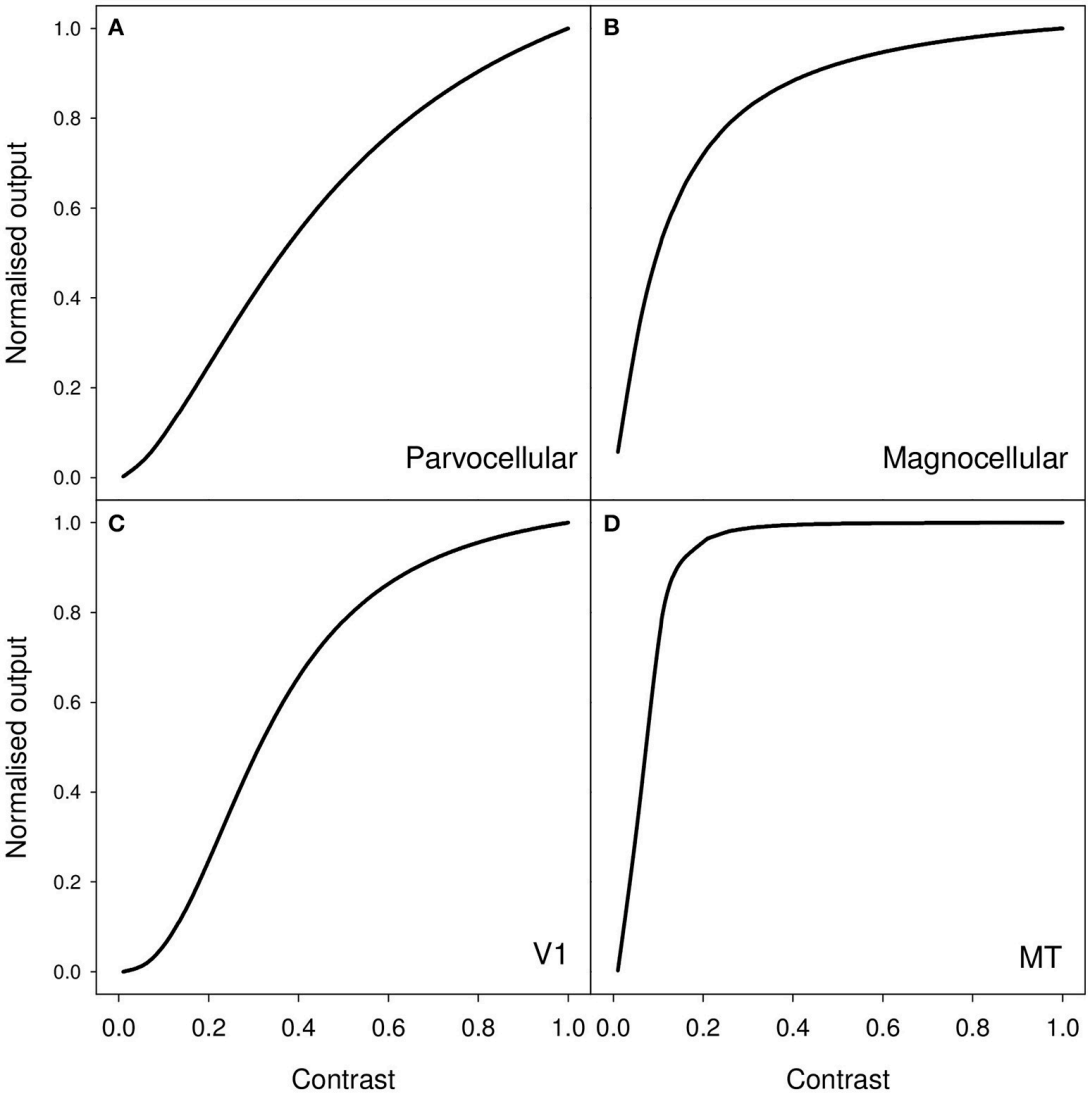
**Representative contrast response curves for the following sets of neurons in macaque (A)** parvocellular LGN, **(B)** magnocellular LGN, **(C)** primary visual cortex (V1), and **(D)** middle temporal (MT/V5) area. The functions shown are generated from median values for fits of the Naka-Rushton function to cells in these various populations. These values are taken from Table 1 (p4) of Sclar et al. (1990).

As an example of the latter, measurements of contrast response from cells in macaque visual pathway show a monotonic increase in neural response as contrast increases. Typically, the response may be characterized by an initial quasi-linear portion followed by compressive non-linearity and saturation (Albrecht and Hamilton, 1982). More particularly, contrast responses in LGN, V1, and MT are well modeled by the Naka-Rushton equation (Sclar et al., 1990); and examples of characteristic curve fits for these three areas are shown in Figure 1. It is clear that, as contrast increases, the amount of neural activity (characterized by firing rate) increases. Therefore, a clear and simple prediction, derived from the idea that perceived duration is driven by the level of stimulus-associated neural activity, is that a high contrast stimulus should appear to last longer than a low contrast stimulus.

Surprisingly, there is little work that looks directly at the effect of contrast on perceived duration; the one study that has explicitly set out to do so has received very little attention (Stoyanova et al., 1987). These authors used magnitude estimation to examine the effects of spatial frequency and contrast on perceived duration. They found that increasing contrast led to increases in estimated duration magnitude. However, this occurred only with short duration stimuli (up to 150 ms); they found no effect with longer duration stimuli (ranging from 190 to 1450 ms) such as those used in the current study. More recently, Bruno and Johnston (2010) compared the perceived duration of high and low contrast drifting sinusoidal gratings. Although they found a tendency for the higher contrast stimulus to be perceived as longer, the effect failed to reach statistical significance. Additionally, one study does look at the effect of *chromatic* contrast, finding that a reduction of the visibility of transient (8.3 ms) equiluminant visual stimuli defining the onset and offset of a brief unfilled interval (100 ms), causes a reduction in that interval's perceived duration (Terao et al., 2008). However, this study does not of course manipulate the contrast of the duration-to-be-judged stimulus (given that this is a blank interval).

Contrastingly, there are many studies that have looked at the effect of stimulus luminance on perceived duration (for example Haber and Standing, 1970; Goldstone et al., 1978; Long and Beaton, 1980; Nisly and Wasserman, 1989). In their review of the literature, Nisly and Waserman tabulate 35 such studies (see their Table 1, p. 488), noting that half show an increase of perceived duration with intensity whilst the remainder show the inverse relationship. Even without the clear lack of consensus, it is difficult to know what such studies might say with regards to an effect of contrast on perceived duration. If you place a stimulus in the middle of a screen and change the stimulus's brightness, then you are changing both contrast and luminance; the two are confounded. Any effects could be due to luminance, to contrast, or an interaction between the two. For example, one recent study looked at the duration of luminance decrements presented against a white background and luminance increments presented against a black background (Matthews et al., 2011). The study showed that perceived duration increased as a function of the difference between the static stimulus (a uniform 4.9° square) and its background, irrespective of whether that difference was a luminance increase or a luminance decrease. Whilst this is certainly consonant with an effect of contrast, it could instead be driven by the evident change in luminous flux.

In the following experiments we look at the effect of stimulus contrast on perceived duration. The stimulus that we use for our duration judgments is spatially isotropic and broadband, yet contains only a single temporal frequency. This choice of stimulus sits well within the context of studies that have produced evidence for local duration processing based on the use of dynamic stimuli (Johnston et al., 2006; Johnston, 2010). The motivation for studying the relationship between contrast and duration lies with the idea that level of stimulus-elicited activity can drive perceived duration. More generally, the nature of this relationship constrains models and adds to our wider understanding of duration perception.

## General methods

These methods were applied in tasks in which participants compared the durations of two visual stimuli. Deviations from, and embellishments of, this general description are addressed in the separate method sections for each experiment.

### Participants

The study was conducted in accordance with the Declaration of Helsinki, and had ethical approval from the Faculty of Science ethics committee at the University of Bristol; informed consent was gained from all research participants. Our pool of participants comprised the two authors and seven naïves all of whom had normal or corrected-to-normal vision. Except for the authors, participants were reimbursed for their time. Note that throughout, participant S1 is author AR and participant S5 is author CB. Further, the participant codes, running from S1 to S9, encompass the full pool of participants who took part in our initial pilot tasks (S1–S5), and two (S6 and S7) who performed relatively poorly on an initial screening task (a duration match using identical stimuli). S6 and S7 did not subsequently take part in any of the contrast experiments reported below. We stick with our original experimental nomenclature to maintain the link between our data files and the results described in this paper. Note that only a subset of participants took part in each task, full details are reported with each experiment.

### Apparatus and stimuli

The experiments were conducted in a darkened room using a gamma-corrected CRT monitor Sony G420 with a screen refresh rate of 120 Hz. Screen resolution was 1024 × 768 pixels and viewing distance was 1 m. Stimuli were generated in Matlab using the Psychophysics Toolbox extensions (Brainard, 1997; Pelli, 1997). Throughout this paper, all contrasts are calculated as the Michelson contrast (*C*_*M*_) where:

$$\begin{array}{l} C_{M}=\frac{\left(L_{max}-L_{min}\right)}{\left(L_{max}+L_{min}\right)} \end{array}$$

Where *L*_*max*_ is the maximum luminance in the image, and *L*_*min*_ the minimum and where their mean (and that of the stimuli) is the display's mean luminance (69.9 cd/m^2^).

**Figure 2:**
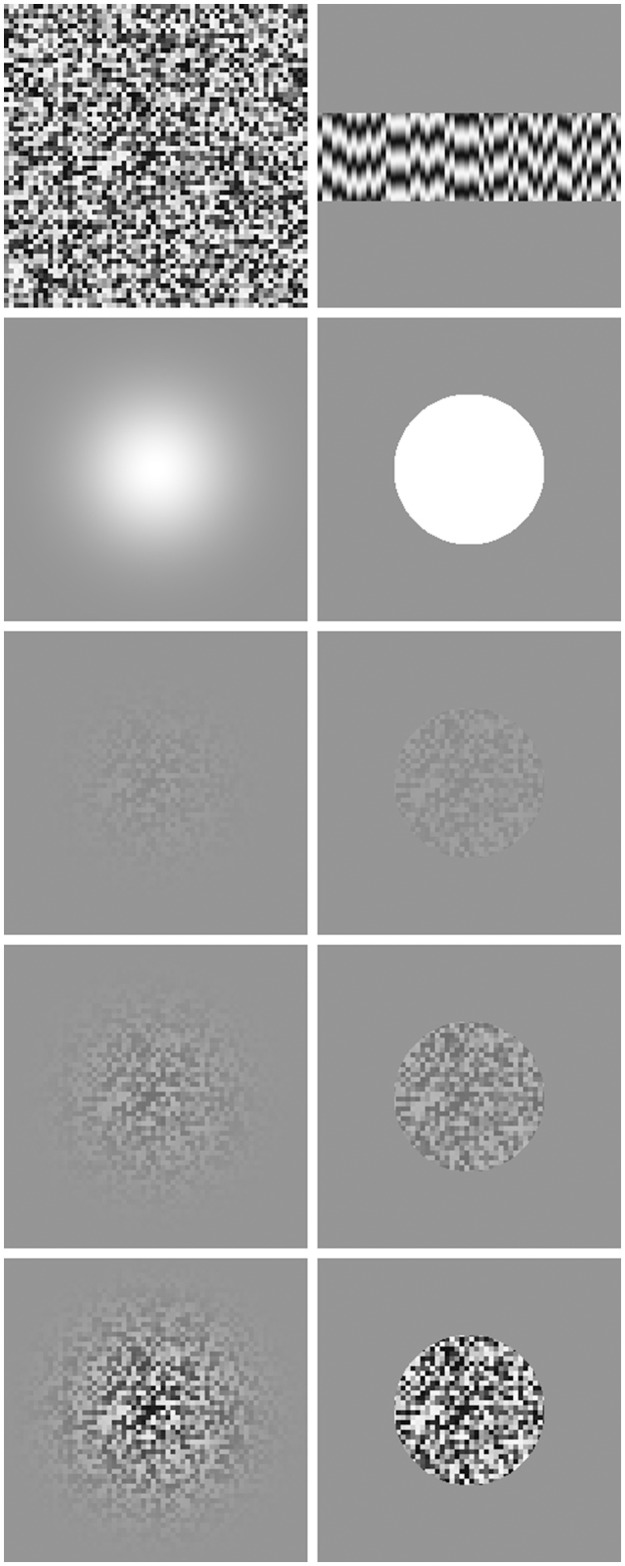
**Stimuli used in our experiments**. Top left shows a single frame prior to being windowed. Top right shows 72 frame long (600 ms) space-time plot, with space along the x-axis and time shown along the y-axis incrementing from top to bottom. Second row shows the masks used to spatially window the stimuli, Gaussian on the left, circular on the right. Remaining images show a single frame at various contrasts (increasing downwards: 0.1, 0.3, and 0.9) for Gaussian windowed stimuli (left) and circular windowed stimuli (right).

Stimuli were composed of patches of spatial noise with the luminance of each noise element temporally modulated at a fixed frequency (commonly, 4 Hz). Noise element size was 4 × 4 pixels (5.8 arc min). Stimuli were windowed in one of two ways, either by a circular Gaussian envelope (standard deviation of 1°) or by a circular aperture (radius of 1.5°). Stimuli were displayed on a gray background set to the mean luminance of the display and were presented perifoveally, their center at 4° to either left or right of a central fixation point. Examples of single frames from our stimuli, along with space-time plots, are shown in Figure 2.

Note that we included the circular window conditions after observing, in pilot work, a contrast-driven difference in apparent size with our Gaussian windowed stimuli. This reflects the fact that, as contrast falls way from the center of a Gaussian windowed stimulus, it reaches threshold more quickly with low contrast stimuli than high contrast stimuli. There is some evidence that larger stimuli appear to last longer (Ono and Kawahara, 2007; Xuan et al., 2007), although this view that has been questioned in more recent work (Yates et al., 2012; Rammsayer and Verner, 2015). If there is an effect of size on perceived duration, then any effect of contrast found with our Gaussian windowed stimulus may reflect the pronounced difference in perceived size, rather than being driven directly by stimulus contrast. The inclusion of the circular window stimulus, which is not affected by this issue of size driven by contrast threshold, allows us to control for this factor and provides an additional set of measures to test for any effect of contrast on perceived duration.

### Design and procedure

Each trial compared a standard with a match stimulus; these were consecutively presented, separated by a randomly-determined interval of between 200 and 400 ms, with presentation side also randomized. The standard stimulus was always presented first, and had a fixed duration (600 ms) and a fixed contrast (0.3). The variable duration match stimulus had a contrast of either 0.9 or 0.1 (match contrasts were randomly interleaved on a trial-by-trial basis). Participants indicated on which side the longer lasting stimulus appeared. We used an adaptive method of constants procedure (Kontsevich and Tyler, 1999) to determine, on a trial-by-trial basis, the duration of the match stimulus. The range of potential match stimuli was constrained to lie between 15 frames (125 ms) and 375 frames (3000 ms) with the adaptive procedure free to choose any whole-number-of-frames stimulus level between those limits. Participant responses generated psychometric functions with the point of subjective equality (PSE) providing an estimate of the duration at which the match stimulus appeared to have the same duration as the 600 ms standard. Each psychometric function comprised 40 trials.

In the following, we use the term *run*, to describe a single psychometric function containing 40 trials and resulting in a PSE. Each experiment contained a number of *blocks*, with each block containing two runs, one with the low contrast match and one with the higher contrast match; these were randomly interleaved on a trial-by-trial basis. Stimulus type (Gaussian or circular windowed) was constant within blocks. Participants repeated each block 4 times, resulting in 4 psychometric functions per match contrast per window type. In each trial, participants indicated which of two stimuli was presented for longer. Responses, given on a hand-held numeric keypad, activated the next trial; a further optional key-strike was available to repeat the last trial before response. Participants were instructed that the emphasis of the task was accuracy rather than speed. Prior to undertaking our duration tasks, each naive participant was instructed to respond only to stimulus duration. Further, each participant undertook a minimum of two training blocks, with each block containing a single run where match contrast was set to the same value as the standard contrast (0.3).

## Analysis

For statistical inference, we rely partly on plotted data along with its associated confidence intervals (Loftus, 1993; Cumming and Finch, 2005). PSEs were estimated by fitting probit functions to our psychometric data (Wichmann and Hill, 2001a). Note that these were fitted as a function of log time; but, for ease of interpretation all graphs are expressed in terms of linear time. To estimate statistical variability of the samples, 95% confidence intervals were calculated using parametric bootstrapping (Wichmann and Hill, 2001b), with 10,000 bootstrap replications per psychometric function. Confidence intervals were calculated using the percentile method (Efron and Tibshirani, 1993). To calculate the confidence for our mean PSEs, and for the differences between these, we run our bootstrap populations through the various averaging and differencing procedures as appropriate (Benton et al., 2006, 2007).

When analysing our data, a small number of our psychometric functions appeared little better than chance. We formalized this observation by applying a model selection technique to our psychophysical data (Skinner et al., 2013; Kent et al., 2014). In addition to our probit function, with its two parameters (location and spread), we employed an additional random button press model. This latter had no parameters; it is simply that at every stimulus level, participants are equally likely to produce either response.

For model selection we used the Akaike Information Criterion (AIC). This is a measure that incorporates the likelihood of the results given the model as well as the number of model parameters, a penalty being applied for the latter. In this scheme, model selection works by comparing AICs with the lower AIC being preferred. As the AIC difference becomes larger then it becomes less likely that the non-preferred model provides a suitable description of the data. The standard guidance for interpreting AIC differences points to an absolute difference of 2 or less in a comparison between two models as providing substantial evidence for both models (Burnham and Anderson, 2002). We wanted reasonable evidence that there was not substantial evidence for our random response model; we therefore chose an AIC difference of 2 as providing enough evidence to allow us to drop the random response model and accept our probit model.

**Figure 3:**
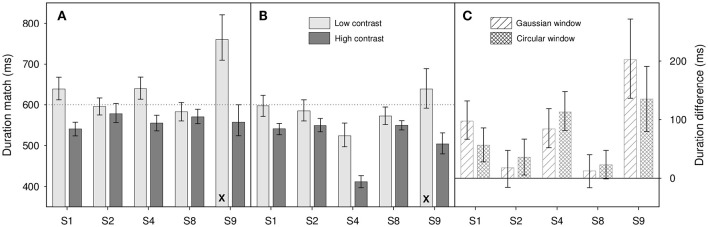
**Results from Experiment 1 for (A)** Gaussian windowed stimuli, **(B)** circular windowed stimuli, **(C)** duration differences for both. Light gray bars show duration matches for the low contrast (0.1) stimuli, dark gray bars show matches for high contrast (0.9) stimuli. **(A,B)** are shown on the same ordinate scale. Diagonally hatched bars show duration differences for Gaussian windowed stimuli, cross-hatched bars show duration differences for the circular windowed stimuli. Differences are calculated as *D*_*low*_ – *D*_*high*_ where *D*_*low*_ is the low contrast duration match and *D*_*high*_ is the high contrast match. Note that the X at the bottom of a bar shows that one psychometric function has been dropped from the analysis (see text for details), those bars therefore show the average of 3 functions, rather than 4. Error bars show 95% confidence limits.

**Figure 4:**
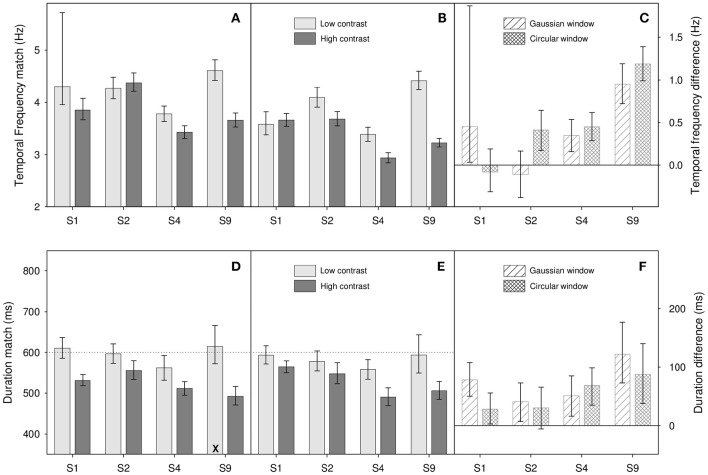
**Results from Experiment 2 for (A,D)** Gaussian windowed stimuli, **(B,E)** circular windowed stimuli, **(C,F)** temporal frequency/duration differences for both. Top panels **(A–C)** show temporal frequency match results, bottom panels **(D–F)** show duration match results. Light gray bars show matches for the low contrast (0.1) stimuli, dark gray bars show matches for high contrast (0.9) stimuli. Diagonally hatched bars show temporal frequency/duration differences for Gaussian windowed stimuli, cross-hatched bars show differences for the circular windowed stimuli. Error bars show 95% confidence limits.

Of the 144 duration judgment psychometric functions gathered in the following experiments, only 3 failed our AIC test (all from participant S9 with low contrast stimuli). Where this occurs, these data have been removed from the analysis; the results shown comprise the mean of the remaining psychometric functions. The removal of a single psychometric function is marked on our bar charts by the use of an X symbol at the bottom of a bar (see Figures 3, 4).

## Experiment 1: an effect of contrast?

Five participants (S1, S2, S4, S8, and S9) first completed all four blocks with the Gaussian windowed stimulus and then repeated these but using the stimulus with the circular window. Note that there are well established order effects in perceived duration. For example, when two stimuli are identical, the second of the pair is usually perceived to be the shorter (Jamieson and Petrusic, 1975). Further, when the second of the pair is different from the first, and the first is a repeated fixed standard whilst the second is a variable match, you might reasonably expect the second to be perceived as longer by dint of the oddball effect (Tse et al., 2004). Because of these various potential order effects, we cannot trust the direct match to the standard to assess whether or not there is a contrast effect; rather, we need to compare the matches of the low and high contrast stimuli to that standard. If we expect an increase in contrast to be associated with an increase in perceived duration, then we should see a *decrease* in the match-to-standard duration of our high contrast (0.9) stimulus relative to the match-to-standard duration of our low contrast (0.1) stimulus.

Results are shown in Figure 3. Where differences are calculated (Figure 3C) we take the difference in perceived duration between the 0.1 contrast and 0.9 contrast match stimuli. This means that an increase in perceived duration with increased contrast is coded positively.

Looking at results across participants, there does appear to be an effect of contrast on perceived duration within increases in contrast causing an increase in perceived duration. This is best illustrated in Figure 3C where we show the differences in duration match between the low and high contrast match stimuli. All 10 differences show a bias in the same direction with many of the individual participant results showing a significant difference from zero (indicated by the zero line lying outside their error bars). This observation is confirmed by a two-factor repeated-measures ANOVA on log duration which shows a significant main effect of contrast [*F*_(1, 4)_ = 8.88, *p* = 0.041], a marginal effect of window type [*F*_(1, 4)_ = 4.93, *p* = 0.091] and no significant interaction between the two [*F*_(1, 4)_ = 0.006, *p* = 0.94].

One possibility that we need to consider is that the contrast-duration effect is caused not directly by contrast, but instead by an effect of contrast on perceived flicker. There is evidence that contrast affects perceived flicker (Thompson and Stone, 1997; Hammett and Larsson, 2012). For example, Thompson and Stone (1997) found that decreasing the contrast of 4 Hz sinusoidal gratings increased their perceived flicker rate. Conversely, Hammett and Larsson (2012) found that at 4 Hz, decreasing contrast decreased perceived flicker speed. Hammett and Larsson explained their failure to replicate Thompson and Stone's (1997) findings as potentially attributable to differences in stimulus parameters.

If our stimuli follow the pattern described by Hammett and Larsson (2012) we would expect our low contrast stimuli to have a lower apparent temporal frequency than our high contrast stimuli. This is potentially problematic because increasing the temporal frequency of a visual event has been found to correspond with an increase in its apparent duration, an effect that saturates at 4–8 Hz (Kanai et al., 2006). It is possible that our effect of contrast on duration is actually instead an effect of apparent flicker on perceived duration. In the following experiment we control for this possibility.

## Experiment 2: controlling for differences in perceived flicker

In this experiment, four participants (S1, S2, S4, and S8) first completed temporal frequency match tasks. We then fed the results of these into our duration match tasks in order to adjust for individual differences in perceived temporal frequency. A similar procedure has been used to equate for difference in perceived speed in other studies (Burr et al., 2007; Latimer et al., 2014). To create the frequency match task we simply took our original duration match task, set the duration of the standard and match stimuli to 600 ms, kept the temporal frequency of the standard at 4 Hz, and allowed our adaptive procedure to control the temporal frequency of the match stimulus. Rather than “longer” or “shorter” than the standard, participants responded “slower flicker” or “faster flicker” than the standard. In terms of number of blocks, number of trials per block, two stimulus types (Gaussian/circular window) and so on, the temporal frequency task is an exact copy of the previous duration match experiment. We then repeat the previous duration match experiment but with the temporal frequencies of the match stimuli set to perceptually match the perceived temporal frequency of our 4 Hz standard stimulus.

Figure 4 shows results of the temporal frequency (Figures 4A–C) and duration tasks (Figures 4D–F). As can be seen from Figure 4F, and as in the previous experiment, there appears to be an effect of contrast on perceived duration, with higher contrast stimuli appearing to last longer. This observation is supported by a two-factor repeated-measures ANOVA on log duration which shows a significant main effect of contrast [*F*_(1, 3)_ = 17.6, *p* = 0.025]. Additionally, we find a non-significant main effect of window type [*F*_(1, 3)_ = 0.82, *p* = 0.43] and a non-significant interaction [*F*_(1, 3)_ = 1.46, *p* = 0.31]. In terms of the effect of contrast on perceived duration, our results tell a consistent story; perceived duration is affected by contrast. As a rough ballpark figure, our reduction in contrast from 0.9 to 0.1, an 89% reduction in stimulus magnitude, resulted in a reduction of perceived duration of about 10%.

We can think of this contrast effect consisting of two components. Firstly, duration compression as a consequence of the reduction in sustained neural activity associated with the stimulus. Secondly, duration compression as a consequence of differences in perceived stimulus onset and offset resulting from the different contrasts. A contrast-driven difference in, for example, perceived onset could well result by proposing that onset is driven by some threshold of neural activity being passed. A reduction in contrast will lead to a slower rise, and faster fall, to a fixed threshold, thereby causing a reduction in perceived duration.

Within the duration literature, one way that has been used to separate effects of onset/offset vs. that of sustaining activity has been to measure perceived duration at a number of standard durations. The logic here is that a distortion in perceived duration based on a shift in onset/offset latencies will manifest as an absolute shift in perceived duration. In contrast, if such a distortion is based on the level of activity during stimulation, then we would see a proportional shift in perceived duration. However, this makes the assumption that shifts in offset latency are not dependent upon the length of stimulation preceding offset. This view does not take account of dynamic processes that occur during visual processing and result in, for example, adaptation driven suppression of neural activity (Clifford et al., 2007). Consequently, in the following experiment, we measure onset and offset directly.

## Experiment 3: onset and offset matching

To create our onset/offset tasks, we adjusted our original duration comparison task as follows. Rather than presenting two stimuli per trial we presented only one; side randomly determined. To provide the temporal comparison with onset or offset we used a polarity reversal of the fixation point. Our original fixation point was a black spot (diameter of 8 pixels) surrounded by a white annulus (band width of 3 pixels). We reduced the luminance of the annulus so that the mean luminance of the fixation spot was equal to the display mean luminance. We then contrast inverted this fixation to produce a second version of our mean luminance fixation spot. For our temporal comparison signal we simply used a contrast inversion of the fixation spot, replacing the current fixation with its opposite polarity version. The motivation for using a single state change for our temporal comparison was that we wanted to use a stimulus with an obvious temporal change but with no duration, at least not within the context of a single trial.

In our onset/offset task we used only Gaussian windowed stimuli. We employed a standard method of constants (MOC) procedure with 10 stimulus levels (25, 67, 108, 158, and 200 ms before and after onset/onset), each presented 10 times. With our two contrast levels (0.9 and 0.1) this gives us 200 trials per block (random presentation order). Each participant completed 4 blocks of onset trials and 4 blocks of offset trials. We used a standard MOC procedure instead of our earlier adaptive procedure because we wanted to ensure that there were enough easy comparisons in the mix of stimuli shown.

**Figure 5:**
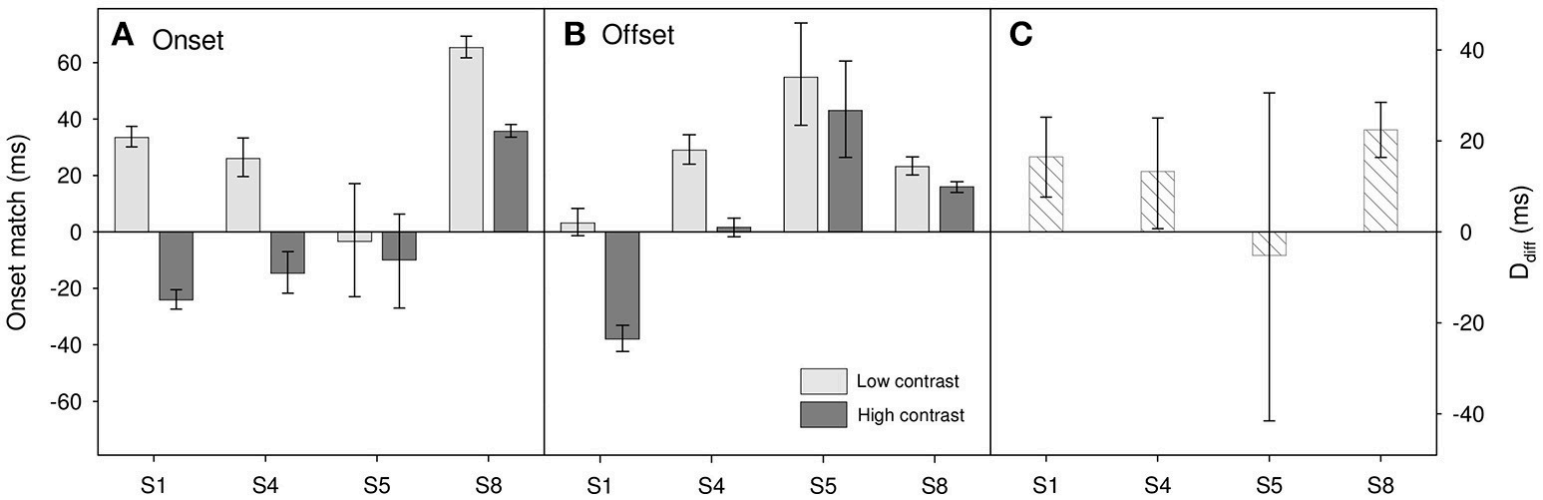
**Results for Experiment 3 for (A)** onset matches and **(B)** offset matches. Light gray bars show matches for low contrast (0.1) stimuli, dark gray bars show matches for high contrast (0.9) stimuli. **(C)** Onset/offset resultant duration difference, *D*_*diff*_ (see text for details). Error bars show 95% confidence limits.

Results for the four participants (S1, S4, S5, and S8) who took part in this task are shown in Figure 5 with the topmost panel showing onset matches and the middle showing offset matches. Positive values show that the polarity change of the fixation needed to be delayed relative to actual stimulus onset in order to appear to match. For each contrast level we can calculate the onset/offset driven change in perceived duration as:

$$\begin{array}{l} {\text{D}}_{0.1}={\text{offset}}_{0.1}-{\text{onset}}_{0.1} \end{array}$$

and

$$\begin{array}{l} {\text{D}}_{0.9}={\text{offset}}_{0.9}-{\text{onset}}_{0.9} \end{array}$$

This makes obvious sense, an increase in offset increases duration, an increase in onset decreases it. We then calculate the onset/offset driven difference in perceived duration between the two contrast levels as:

$$\begin{array}{l} {\text{D}}_{\text{diff}}={\text{D}}_{0.9}-{\text{D}}_{0.1} \end{array}$$

A two-factor repeated-measures ANOVA on onset-offset match shows a marginally significant effect of contrast [*F*_(1, 3)_ = 9.83, *p* = 0.052], but no significant effect of match type [onset vs. offset, *F*_(1, 3)_ = 0.02, *p* = 0.89] and no significant interaction [*F*_(1, 3)_ = 3.92, *p* = 0.14]. This indicates the possibility that, as expected, perceived onset and perceived offset may be comparatively delayed in the lower contrast stimulus. When looking at the subject-by-subject data (Figure 5C) there does appear to be an onset/offset driven difference for three participants (S1, S4, and S8) with the perceived duration of the lower contrast stimulus having the shorter duration. However, any such effect does appear small. A reasonable upper estimate would be a ~20 ms difference; too small to account for the ~60 ms difference that we find in perceived duration. So whilst we cannot rule out the idea that onset/offset differences may play a part, it would seem unlikely that they can account for the entire contrast-duration effect.

## Modeling contrast-driven response bias

Given the small size of our contrast-duration effect, it is worth considering whether participants, when unsure of a duration judgment, might use the clear difference in contrast (between standard and test) to determine their response. If participants tended to respond “longer” for higher contrast stimuli then this would tend to make these stimuli appear to last longer, at least in the eyes of our measurement technique. Similarly, a tendency to respond “shorter” for lower contrast stimuli would reduce measured duration.

Faced with this possibility, one course would be to ignore the issue. As the reader will see, the points that we make in our General Discussion are based on the modest size of our contrast-duration effect. If we simply accept that contrast-bias may be a factor, then our results provide an upper estimate of the contrast effect, which leaves the conclusions drawn in our discussion unchanged. An alternative is to use a variety of different experimental techniques and assume that this will somehow resolve the issue—this is more likely to present a confusing scenario in which some techniques drastically overestimate the effect, whilst others hide any effect in measurement noise.

Instead, in the following we develop a model of response bias and then use a model selection technique to see if this provides a better account of our data than a contrast-duration effect. We assume that participant responses are characterized by an underlying psychometric function modeled by the integral of the normal distribution (with mean μ and standard deviation σ*)* as a function of log_*e*_ duration (*t*_*e*_):

$$\begin{array}{l} F\left(t_{e}|\mu ,\sigma \right)\;=\;\frac{1}{2}\left[1+erf\left(\frac{t_{e}-\mu }{\sigma \sqrt{2}}\right)\right] \end{array}$$

where *F*(*t*_*e*_ | μ, σ) is the expected proportion of “longer” responses at log duration *t*_*e*_.

We can conceptualize the responses at any level of *t*_*e*_ as being made up of a proportion of certain responses, and uncertain responses, where these sum to unity. When the difference between *t*_*e*_ and μ is large (as a proportion of σ) then the duration judgment is unambiguous, however when *t*_*e*_ is equal to μ then the participant is uncertain of their response. We can calculate the proportion of certain responses as follows:

$$\begin{array}{l} C\left(t_{e}|\mu ,\sigma \right)\;=\;2|F\left(t_{e}|\mu ,\sigma \right)-\frac{1}{2}| \end{array}$$

and the proportion of uncertain responses as:

$$\begin{array}{l} U\left(t_{e}|\mu ,\sigma \right)\;=\;1-C\left(t_{e}|\mu ,\sigma \right) \end{array}$$

So, for example if, in the absence of bias, we would respond “longer” 80% of the time (at a particular level of *t*_*e*_), then we can think of this as being comprised of a proportion of certain responses (60%) to which we always respond “longer,” with the remainder being uncertain responses, to which, on average, we would ideally respond “longer” half the time.

We introduce bias (β) by characterizing the proportion of uncertain responses in which participants respond “longer” as:

$$\begin{array}{l} P\;=\;\left(\frac{1+\beta }{2}\right) \end{array}$$

where β can range from −1 (where all uncertain responses are “shorter”) through 0 (where responses are unbiased) to +1 (where all uncertain responses are “longer”). In contrast, for responses that are certain, the direction of response is determined by whether *t*_*e*_ is greater than μ or less than μ. When greater, the response is always “longer,” when less, the response is “shorter.” We can characterize this by the following step function:

$$\begin{array}{l} D\left(t_{e}|\mu \right)\;=\;\left\{\begin{array}{c} 0\;if\;t_{e}<\mu \\ 1\;if\;t_{e}\geq \mu \end{array}\right\} \end{array}$$

Note that when *t*_*e*_ = μ then the value of *D* (*t*_*e*_ | μ*)* is immaterial, as at this point, all responses are uncertain.

From the various equations described above we can create our biased psychometric function:

$$\begin{array}{l} B\left(t_{e}|\mu ,\sigma ,\beta \right)=C\left(t_{e}|\mu ,\sigma \right).D\left(t_{e}|\mu \right)+U\left(t_{e}|\mu ,\sigma \right).\left(\frac{1+\beta }{2}\right) \end{array}$$

which takes three parameters, these being the mean and standard deviation of the baseline function (μ, σ) and the response bias (β).

**Figure 6:**
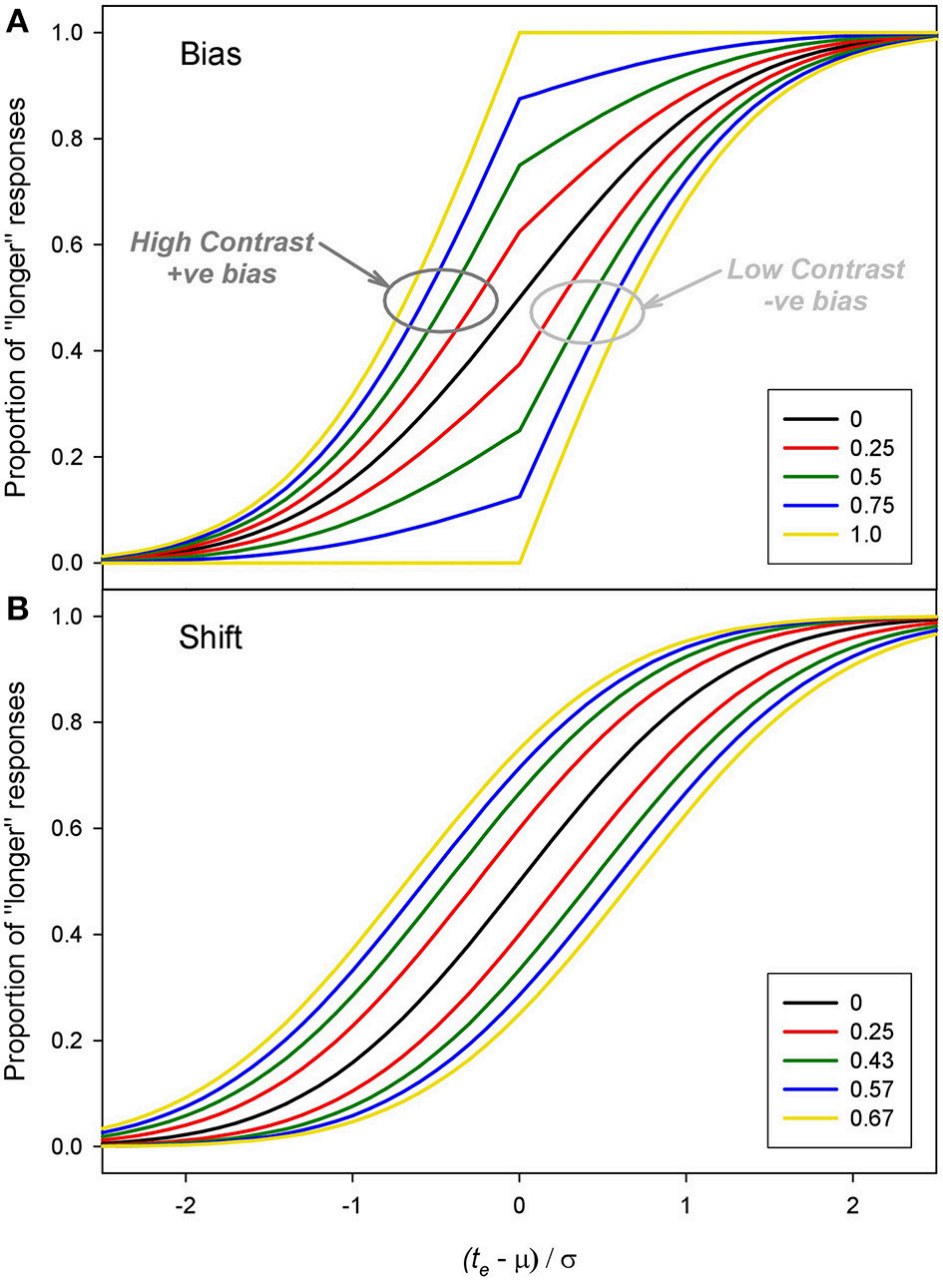
**Examples of pairs of functions with (A)** equal and opposite biases (β) and **(B)** equal and opposite shifts in the mean (μ). For these example functions μ = 0 and σ = 1. The values for the shifts of μ were chosen to produce a similar PSEs to the bias values.

Examples of positive and negative bias are shown in Figure 6A. The effect of bias is to distort, sharpen and shift the underlying psychometric function with positive bias shifting the function leftwards (and negative bias shifting it rightwards). For comparison Figure 6B shows the result of positive and negative shifts in the mean, μ. Although both changes in β and μ can result in measured duration changes, there is a clear difference in the shape of the curves which becomes more obvious with higher magnitudes of bias.

Whilst such a difference would be difficult to pick out from individual psychometric functions, a model fitting analysis applied to a large corpus of psychometric functions may well allow us to decide whether bias or shift is the best way to account for the data. Our participants completed interleaved pairs of runs, one with the test at a low contrast (***B***_*low*_), one with the test at a high contrast (***B***_*high*_). Across participants and duration tasks (so the Gaussian and circular window tasks in both Experiments 1 and 2) we have a total of 72 pairs of complementary psychometric functions. For the following analysis we discarded three of these pairs, these being the little-better-than-chance functions described earlier.

For each interleaved pair we fitted the models described in Table 1. So for example, in Model 3, there is no bias (because β = 0 for both functions) and the low and higher contrast stimuli have underlying psychometric functions that are shifted sideways with respect to one another indicating a shift in the underlying psychometric functions (consonant with a difference in perceived duration). By contrast, in Model 2, the low and high contrast functions have the same underlying psychometric function (indicating no difference in perceived duration) yet opposite but equal biases (−β and +β).

**Table 1:** **Description of models and AIC differences (ΔAICs)**.

**Model**	***B_*low*_***	***B_*high*_***	**Parameters**	**ΔAIC**
1	β = 0, *μ, σ*	β = 0, *μ, σ*	2	271
2	−*β, μ, σ*	*β, μ, σ*	3	24
3	β = 0, μ_1_, σ	β = 0, μ_2_, σ	3	0
4	β_1_, *μ, σ*	β_2_, *μ, σ*	4	40
5	−*β, μ*_1_, σ	*β, μ*_2_, σ	4	112
6	β_1_, μ_1_, σ	β_2_, μ_2_, σ	5	145

*Text in columns 2 and 3 shows the various parameters fitted to the low (**B**_low_) and high contrast functions (**B**_high_). For AIC differences, the AICs for all 69 pairs of psychometric functions are first summed for each model. This results in a set of summed AICs, the differences are then calculated by taking the minimum of the set from each member of the set*.

We fitted a variety of models (see Table 1) to our participants' data by minimizing deviance (so maximizing likelihood) using Matlab's fminsearch function. As before, we used the AIC for model selection. Recall that lower AICs are preferred. The usual method of comparison is to calculate the AIC differences (ΔAICs) between each model and the model with the lowest AIC (see Table 1) with a difference of 10 or more leading us to discount the higher scoring model (Burnham and Anderson, 2002).

The critical analysis shown in Table 1 is between Models 2 and 3 (the bias and shift models) where we see a difference of 24 with the shift model (Model 3) having the lowest AIC and therefore being the most preferred. This AIC difference falls well above the threshold difference of 10 that Burnham and Anderson characterize as providing essentially no empirical support for the least preferred model (see their page 170). We also fitted a number of more complex models (Models 4–6) some of which incorporated differences in both bias and shift (Models 5 and 6). The added complexity bought no advantages, with these models provide a relatively poor description of our data. Further, we also included a simple model (Model 1) in which there is no difference between the low and high contrast functions—essentially, the null hypothesis. In support of our analyses presented with Experiments 1 and 2, this model offers a very poor description of our data. Overall, our simple shift model is the clear winner. These results indicate that our findings are highly unlikely to arise from a simple contrast-based response bias; instead our analysis supports the notion that we are describing an effect of contrast on perceived duration.

## Discussion

Our primary finding is that the perceived duration of a stimulus decreases as its contrast is reduced. This decrease is ~60 ms with a 600 ms standard, so a 10% duration compression with a reduction in contrast from 0.9 to 0.1; or, put another way, our 89% reduction in contrast resulted in a 10% reduction in perceived duration. We can think about this duration reduction of being composed of two components, an onset/offset effect and a sustained activity effect. We certainly cannot rule out some part of the duration effect resulting from onset/offset differences, however at best this would only account for ~20 ms of the duration difference.

A reasonable question to ask is whether the contrast differences that we employ in our experiments should lead to substantial differences in level of neural activity in early visual processing areas. Further, if level of neural activity in early visual areas drives perceived duration, then is the small duration difference that we see a reasonable reflection of the expected differenced in level of neural activity? Based on the contrast response curves shown in Figure 1, we should expect our reduction in contrast to result in a substantial reduction in neural activity in early visual areas. If we simply compare the 0.9 and 0.1 points on each of these curves shown in Figure 1, our 89% reduction in contrast leads to a 90% reduction in estimated activity for the parvocellular LGN neurons, a 49% reduction for the magnocellular neurons, a 94% reduction for V1 neurons and a 26% reduction for MT neurons.

Of course the analysis presented above is a very rough and ready assessment which fails to take account of the nature of our stimulus which will be far from optimal for driving, for example, cells in V1 and MT. For these areas the effective contrast of our stimuli will be lower than the simple Michelson contrast that we use to characterize our stimuli. For example, it is well known that stimuli without coherent motion, such as our flicker stimulus, are less effective at driving the human homolog of macaque MT than coherent motion stimuli (Braddick et al., 2001). A more detailed assessment of effective contrast can be found in Supplementary Materials where we apply filtering operations characteristic of cells in LGN and primary visual cortex to our stimuli (Young and Lesperance, 1993; Benton and Johnston, 1997; Tadmor and Tolhurst, 2000; Benton, 2004).

It is likely then that our low contrast stimulus will produce substantially less activity that that indicated by simply reading the 0.1 point off the curve shown in Figure 1D. The 26% reduction for MT cells that we calculate above is likely to substantially underestimate the true size of any contrast-driven reduction in neural activity. Further, and as shown in our computational assessment in Supplementary Materials, our stimulus is far from optimal for driving oriented receptive fields such as those found in primary visual cortex. In contrast, with its lack of oriented structure, the stimulus proves rather effective at driving receptive fields characteristic of those found in LGN (see Supplementary Materials).

In general, we therefore have good reason to believe that, with our stimulus, there will be a substantial difference in the magnitude of neural activity associated with the stimulus in LGN, V1 and MT. Further, the stimulus is likely to produce substantial activity in LGN with decreasing activity found as one moves up the visual system and receptive fields become more elaborate.

Our modest contrast effect is problematic for the idea that the level of stimulus associated neural activity in early visual areas drives perceived duration. This difficulty comes from considering our results in the context of a recent study looking at repetition suppression and the oddball effect (Sadeghi et al., 2011). Recall firstly, that the oddball effect is the expansion of apparent duration of a novel stimulus presented within a train of repeated stimuli; and secondly, that repeated presentation of a stimulus suppresses the neural response associated with that stimulus (Li et al., 1993; Grill-Spector et al., 2006). This relationship has been used to motivate the idea that the level of stimulus-related neural activity can drive the perceived duration of that stimulus (Pariyadath and Eagleman, 2012).

Sadeghi et al.'s (2011) study is particularly interesting because they look at the oddball effect using both human psychophysics and macaque electrophysiology. The stimulus they used, a random dot kinematogram, is a standard stimulus for driving neurons in motion area MT. In one condition, they used fixed length sequences of 6 pulses, the last of which was either an oddball or a repeat of the previous stimuli. In order to match the previous set of stimuli (all 200 ms), the duration of the oddball had to be reduced to ~160 ms, whereas the duration of the repeat had to be increased to ~310 ms. For purpose of comparison, we can quantify the magnitude of this effect by taking the difference and dividing by the mean; which gives an effect magnitude of ~64%. Note that these figures, and those given below, are carefully estimated from Figures 1, 2 of Sadeghi et al. (2011).

In stark contrast, the averaged normalized neural response, calculated by taking the difference between neurons tuned to the optimal and opposite directions, was ~1.07 for the oddball and ~0.95 for the repeat, an effect magnitude of ~12%. Sadeghi et al. (2011) note the discrepancy and appeal to a number of possible explanations, however the difference in effect magnitude is substantial and is difficult to reconcile with the fact that our 89% reduction in contrast, predicted to result in a similar reduction in neural activity, resulted in, at best, a 10% reduction in perceived duration.

Clearly, if level of stimulus related neural activity drives perceived duration, we should expect larger differences in neural activity to result in larger differences in perceived duration. This idea cannot readily explain how our large contrast-driven difference in expected neural activity results in only a small difference in perceived duration—at least, not when smaller changes in neural activity found with other manipulations results in larger changes in perceived duration. Taken in combination, these findings point to a dissociation between level of stimulus-related neural activity in early visual areas and perceived duration.

Our findings imply that, for models of duration processing that rely on the temporal integration of stimulus-related activity, if these models are correct, then the activity upon which they draw must be from brain areas showing contrast invariant neural responses. Of course, it may well be the case that these models are incorrect and that we must look to other approaches such as state dependent networks (Buonomano and Maass, 2009), duration tuned channels (Heron et al., 2012) or gradient-based models to understand duration perception (Johnston, 2010).

However, we would like to stress that our results do not necessarily imply that mechanisms in early visual processing do not contribute to perceived duration. Changes in the level of neural response are not the only consequence of changes in contrast. For example, there is good evidence that rapid contrast gain in magnocellular retinal ganglion cells results in a shortening of their temporal impulse response function (Shapley and Victor, 1978; Benardete et al., 1992; Lee et al., 1994; Benardete and Kaplan, 1999; Stromeyer and Martini, 2003). This means that the cells become tuned to higher temporal frequencies and their peak response advances (so with less delay between stimulus onset and peak response). Is it possible that effects such as these underlie the duration distortion described in the current study?

One group of researchers have proposed a link between contrast gain and perceived duration. Bruno and Johnston (2010) showed an effect of rapid contrast adaptation on perceived duration which they take to indicate just such an influence of early magnocellular temporal filtering operations in perceived duration. Further, the same authors also demonstrate that substantial reductions in background luminance both lengthen the temporal impulse response function, and lead to an expansion of perceived duration (Bruno et al., 2011). Again, they tie this to a scaling of the temporal impulse response function early in visual processing (Kelly, 1961; Purpura et al., 1988).

However, Bruno and Johnston found temporal compression of their 50% contrast stimulus following high contrast adaptation (90%) in comparison to low contrast adaptation (10%). Contrastingly, we found duration expansion with our higher contrast stimulus in comparison to our low contrast stimulus. Further, Bruno and Johnston only found their effect at higher temporal frequencies (≥10 Hz), our stimuli were presented at 4 Hz. Additionally, when directly comparing the perceived duration of high and low contrast stimuli Bruno and Johnston found no significant effect of contrast on perceived duration, both at 5 and 10 Hz. Clearly, the picture is unclear, and there is much to be resolved in the relationship between contrast and perceived duration. At present, the idea of a low level mechanism based on magnocellular processing remains an intriguing possibility.

In conclusion, we found only a small effect of contrast on perceived duration with a reduction in contrast leading to duration compression. The small size of our contrast effect needs to be reconciled with the substantial shifts in perceived duration that have been found with the oddball effect; especially given that these seem associated with much smaller changes in neural activity than we might reasonably ascribe to our contrast difference. This implies that there is no simple monotonic relationship between general level of neural activity in early visual areas and perceived duration.

## Author contributions

CB and AR designed the research, AR carried out data collection, CB analyzed the data and wrote the paper.

### Conflict of interest statement

The authors declare that the research was conducted in the absence of any commercial or financial relationships that could be construed as a potential conflict of interest.
