# Supplementary material for: Perceived Duration Increases with Contrast, but Only a Little
Source: Front Psychol. 2016 Dec 15;7:1950. doi: 10.3389/fpsyg.2016.01950 (PMC5156709; doi:10.3389/fpsyg.2016.01950)
Supplement: Supplementary file 1 [file Image1.pdf]

Supplementary Material: Perceived duration increases with contrast, but only a little.

Christopher P Benton and Annabelle S Redfern
School of Experimental Psychology, University of Bristol, UK.

Computational assessment of stimulus-driven neural responses

In order to assess how changes in contrast might affect changes in neural activity (and any consequent effects on perceived duration), it would be useful to have some idea of how much neural activity might change in response to our change in contrast, in particular in regard to the two match stimulus contrast levels (10% and 90%) used throughout our experiments. In Figure 1 of our paper, we showed representative examples of contrast response curves for LGN, V1 and MT. However, the neural responses measured to derive these curves were gathered in response to sine wave stimuli; in particular to stimuli at the optimal (or close to optimal) set of parameters to drive those neurons investigated. In contrast, our stimuli are spatially stochastic and are comprised of small 4x4 pixel patches, the luminance of each of which is temporally modulated sinusoidally. Such stimuli are clearly less than ideal for, for example, driving simple cells in primary visual cortex.

If we want to understand how the contrast response curves shown in Figure 1 might apply to our stimuli, we need to assess the activity that would be elicited with our stimuli in comparison to the standard sinusoidal stimuli commonly used to measure contrast response curves. In Figure S1 we show the results of applying various spatial filters to a single frame from our stimulus. Note that we ignore the temporal domain as our stimulus is temporally sinusoidal; the response-attenuating characteristics lie in the spatial domain. The filters were chosen to be representative of spatial receptive fields present in LGN and primary visual cortex. The centre-surround LGN receptive field falls within the midrange of filter parameters used to model LGN responses to images of natural scenes (Tadmor and Tolhurst, 2000). The even and odd Gabor filters are commonly used to model simple cells in primary visual cortex (Benton and Johnston, 1997) - we chose parameters for these such that they provide a close match to Gaussian derivative filters that have also been used to model such responses (Benton, 2004; Young and Lesperance, 1993).

The images shown in Figure S1 are normalised with respect to the maximum output from each filter when applied to an optimal sine wave with the same Michelson contrast. Applying our centre-surround LGN filter results in only a slight blurring of the noise pattern and the range of output magnitudes closely matches that in response to the sine wave. This makes obvious sense as the LGN filter is lowpass and spatially isotropic. In contrast, when we apply our oriented Gabor filters, then we obtain comparatively reduced output. Again, this makes obvious sense, the filter is oriented, our stimulus is spatially isotropic and stochastic.

Whilst viewing the filtered normalised images allow some assessment of filter output, a more finely grained assessment is shown in the bottom panels of Figure S1. The grey bars show the output from our noise stimulus whilst unfilled boxes show the output that results from a sine wave grating. To generate these histograms we produced 100 instantiations of each of our 480x480 pixel images, applied our spatial filters (each with 81x81 pixel support), and collated the resultant 400x400 pixel output images. The output for our LGN filter (a) and even Gabor (b) are shifted to positive values as both of these filters have a DC component. The incongruous leftmost bar for the sine wave responses shown in (b) is a binning artefact (we used the same bin edges for all histograms).

For all three, the pattern of responses with the noise stimulus is very different from that found with

the sine wave grating. There are far fewer extreme values and most of the activity is clustered around lower output values. Certainly, for cell responses in primary visual cortex, the vast majority of expected outputs will be lower than those that would be produced if cell receptive fields were stimulated with an optimal sine wave grating with a quarter of the contrast of our noise pattern. With the LGN cell this is less pronounced but it is clearly true that the majority of the outputs will fall under 0.5 sine wave equivalent contrast.

The simple point here is that, for LGN and V1 neurons responding to our stimulus, we would expect the majority of cell outputs to fall below the level where we might expect cell responses to saturate. With cells in striate cortex, our two contrasts will largely fall within the range of cell responses that shows a quasi-linear relationship between contrast and cell output (Albrecht and Hamilton, 1982). In other words, in primary visual cortex, lowering our contrast from 0.9 to 0.1 should result in a similarly scaled reduction in neural response.

Similarly, for both parvocellular and magnocellular LGN cells, we should also obtain a substantial reduction in population neural activity as a result of our reduction in contrast. A further point to make is that these patterns of results will generalise across a wide range of filter parameters. Indeed, the results of our filtering operations are obvious, at least in retrospect. Centre surround cells respond to differences, particularly to something like a bright spot on a dark background. With our stimulus, by dint of its stochastic nature, such extremes are rare and there are many areas where there may be little local difference between patches. Further, and as noted previously, the comparatively low level of activity associated with orientation selective filters is simply a reflection of the fact that our stimulus is spatially isotropic.

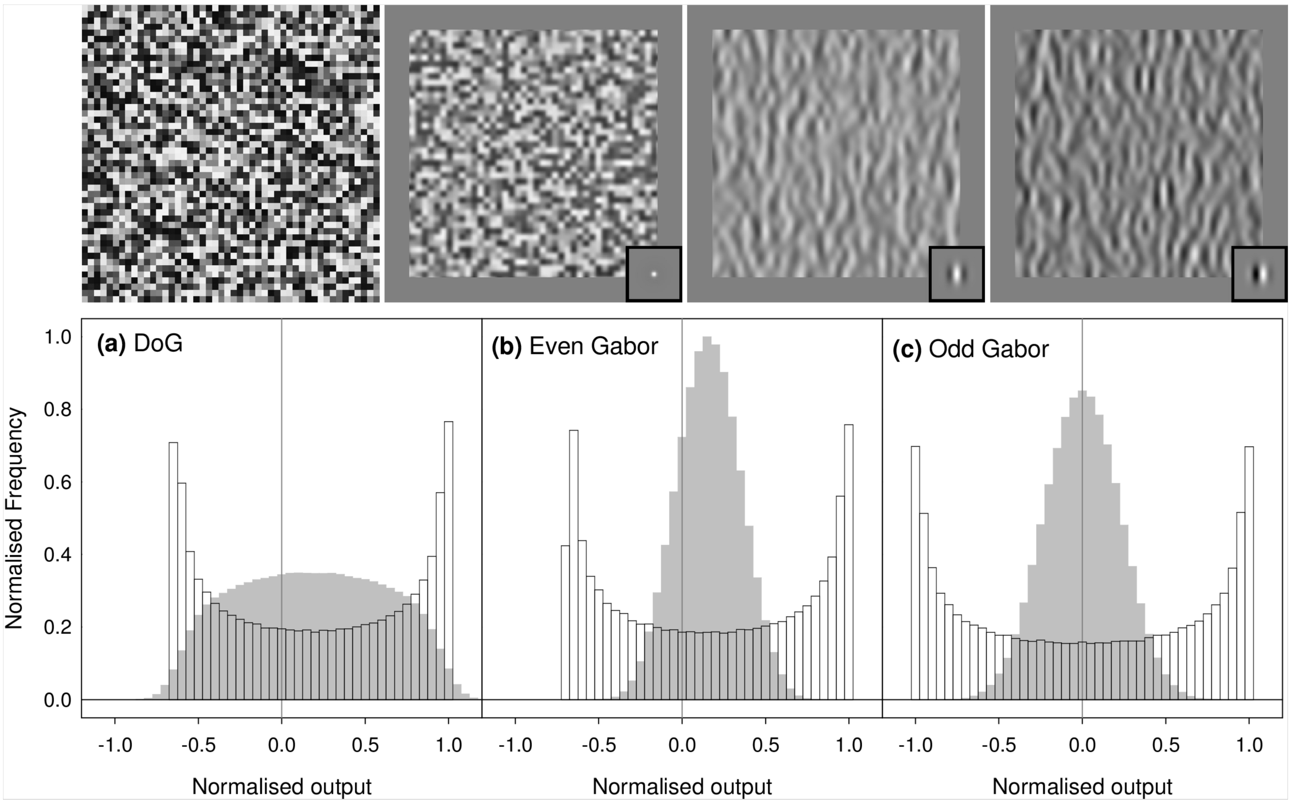

Figure S1: Top row shows, from left to right, (1) a single unwindowed frame, (2) that frame filtered with a difference of Gaussian, (3) filtered with an even Gabor and, (4) filtered with an odd Gabor. Inset at the bottom right of each filtered image shows the filter that was applied. Bottom row shows histograms of filter output for our noise stimulus (solid grey bars) and a sine wave grating (clear bars) for the following (a) difference of Gaussian, (b) even Gabor, (c) odd Gabor. See text for details.

We might also ask what levels of activity we might expect to find as we move up the motion system to area MT. This is an area with large receptive fields that respond well to motion stimuli. The activity of cells in MT is monotonically related to motion coherence with increasing coherence resulting in increasing neural response (Braddick et al., 2001; Britten et al., 1993). Ours is a flicker stimulus, not a motion stimulus; it has no global motion coherence although it will have random patches of coherent local motion. Consequently, we should expect the activity elicited by our low contrast stimulus to fall on the lower non-saturated portion of the MT contrast response curve. This will necessarily result in a substantial difference in neural response between our low and high contrast stimuli.

References

Albrecht, D., and Hamilton, D. (1982). Striate Cortex of Monkey and Cat - Contrast Response Function. *J. Neurophysiol.* 48, 217–237.

Benton, C. P. (2004). A role for contrast-normalisation in second-order motion perception. *Vis. Res* 44, 91–98.

Benton, C. P., and Johnston, A. (1997). First-order motion from contrast modulated noise? *Vis. Res* 37, 3073–3078.

Braddick, O. J., O'Brien, J. M. D., Wattam-Bell, J., Atkinson, J., Hartley, T., and Turner, R. (2001). Brain areas sensitive to coherent visual motion. *Perception* 30, 61–72.

Britten, K. H., Shadlen, M. N., Newsome, W. T., and Movshon, J. A. (1993). Responses of neurones in macaque MT to stochastic motion signals. *Vis Neurosci* 10, 1157–1169.

Tadmor, Y., and Tolhurst, D. J. (2000). Calculating the contrasts that retinal ganglion cells and LGN neurones encounter in natural scenes. *Vision Res.* 40, 3145–3157. doi:10.1016/S0042-6989(00)00166-8.

Young, R., and Lesperance, R. (1993). *A Physiological Model of Motion Analysis for Machine Vision.* , eds. J. P. Allebach and B. E. Rogowitz Bellingham: Spie - Int Soc Optical Engineering.
